# Formation of the mutagenic DNA lesion 1,N^2^-ethenoguanine induced by heated cooking oil and identification of causative agents

**DOI:** 10.1186/s41021-023-00284-3

**Published:** 2023-10-25

**Authors:** Hiroshi Kasai, Kazuaki Kawai

**Affiliations:** Department of Environmental Oncology, Institute of Industrial Ecological Sciences, University of Occupational and Environmental Health, 1-1 Iseigaoka, Yahatanishi-ku, Kitakyushu, Fukuoka 807-8555 Japan

**Keywords:** 1,N^2^-ethenoguanine, 2,4-heptadienal, Cooking oil, Gastrointestinal carcinogenesis

## Abstract

**Background:**

The DNA-damaging compounds in heated cooking oil were identified as guanosine adducts. Heated vegetable oil was subjected to deep-frying conditions at 170 °C for 45 min, reacted with isopropylidene guanosine (ipG) at pH 7.4, and the resulting compounds were separated by high-performance liquid chromatography (HPLC).

**Results:**

Two adducts, 8-hydroxy-ipG and 1,N^2^-etheno-ipG, were identified in the reaction mixture. One of the major components in heated cooking oil, 2,4-heptadienal (HDE), efficiently produced etheno-ipG from ipG in the presence of tBuOOH. An oxidized HDE solution was fractionated using HPLC to identify causative agents, and each fraction was tested for etheno-ipG formation. In addition to the known lipid peroxidation product, 4,5-epoxy-2-heptenal, two unknown polar components with potent etheno-ipG formation activity were discovered. Based on Mass and UV spectra, their structures were identified as 6-oxo- and 6-hydroxy-2,4-HDE. Similarly, 6-oxo- and 6-hydroxy-2,4- decadienal (DDE) were formed from 2,4-DDE. Significant amounts of 6-oxo- and 6-hydroxy-2,4-alkadienal were detected in the heated cooking oil. These compounds induced the formation of 1,N^2^-ethenoguanine in nucleosides and DNA, especially in the presence of tBuOOH. Moreover, the formation of 6-oxo- and 6-OH-HDE from 2,4-HDE was accelerated in the presence of hemin and tBuOOH.

**Conclusion:**

The results suggest that these compounds are not only generated during the oil heating process but also produced from 2,4-alkadienal through digestion under normal physiological conditions, especially after ingesting heme- and alkyl-OOH-containing diets. Moreover, these compounds can be formed within cells under oxidative stress, potentially linking them to gastrointestinal carcinogenesis.

**Supplementary Information:**

The online version contains supplementary material available at 10.1186/s41021-023-00284-3.

## Introduction

Epidemiological studies have consistently shown that diet plays a significant role in developing human cancer [1]. Notably, the consumption of fried food has been associated with an increased risk of various cancers, including esophageal [2, 3], stomach [3–6], colon [7], prostate [8], ovarian [9], and breast [10] cancers. In vitro micronucleus test (S9+, S9-) of deep-fried food revealed genotoxicity [11]. Despite these findings, information about the carcinogens and DNA-damaging agents in fried foods remains limited, except for acrolein and acrylamide, both being classified as Group 2A (i.e., probable human carcinogens) by the International Agency for Research on Cancer. This study sought to identify the cancer-causing agents by extensively investigating guanine modifications induced by heated cooking oil as a model for DNA damage. In the early 1980s, Kasai and Nishimura developed a method to trap direct-acting mutagens as guanine-adducts in a complex mixture of food extracts and model reactions of heat-processed foods. This groundbreaking approach led to the discovery of 8-hydroxyguanine (7,8-dihydro-8-oxoguanine) [12]. This method is based on the concept that many carcinogens tend to react with DNA bases, particularly guanine [13]. This established method was employed in the present study to evaluate the heated cooking oil, as fried foods often contain absorbed heated oil (8–18%, w/w) from the deep-frying process [14]. It was hypothesized that major cancer-causing mutagenic agents could be formed during oil heating. This paper elucidates guanine-adducts’ structure and explores the causative agents, providing a possible link to the development of gastrointestinal carcinogenesis.

## Experimental

### Materials

2,4-heptadienal (90%), 2,4-decadienal (90%), t-butylhydroperoxide (tBuOOH) (70%), hydrogen peroxide (30%), chloroacetaldehyde, and MnO_2_ were purchased from Tokyo Chemical Industry Co., Ltd. Japan. Hemin, 2’,3’-O-isopropylideneguanosine (ipG), and calf thymus DNA were purchased from Sigma-Aldrich Chemical Co., USA. Cooking oil, composed of soybean and rapeseed oil, was purchased from a grocery store. The commercial ipG was purified by semi-preparative high-performance liquid chromatography (HPLC, Shiseido Capcell Pak, C-18,10 × 250 mm), using 30% aqueous ethanol for elution. Authentic samples of 8-OH-ipG and ε − ipG were prepared using the methods described in the literature [12] and [15], respectively. 1,N^2^-ethenoguanin (εGua) was obtained by acid hydrolysis (0.1N HCl, 37 °C, 16 h) [16] of 1,N^2^-etheno-2’-deoxyguanosine as described [15].

### Analysis of ipG-adducts induced by heated cooking oil

Cooking oil (50 mL) was heated at 170 °C for 45 min on a pan with a lid by auto-regulation of temperature. After cooling, the major part of the heated oil was divided into Eppendorf tubes and kept in a freezer at –20 °C. The heated oil (70 µL) was mixed with ipG solution [0.5 mg ipG; 200 µL acetonitrile; 150 µL 100 mM phosphate buffer (pH 7.4)] in a capped Eppendorf tube and reacted at 37 °C in emulsion for 3 d with occasional mixing. Control oil and ipG with the same concentration were also incubated under the same conditions. Each 70 µL of reaction mixture (emulsion) was injected into an HPLC apparatus (Hewlett-Packard 1100 system connected with a photodiode array UV detector) with a column (Shiseido Capcell Pak C-18, 4.6 × 250 mm, 5 µm) at temperature: 23 °C; speed, 0.5 mL/min; elution, linear gradient of ethanol concentration in 10 mM ammonium formate, 0.1% acetic acid: 0–60 min, 4–30%; 60–90 min, 30–77%.

Mass spectrometry of the isolated ipG-adducts by HPLC was conducted using an HPLC (UltiMate 3000, Thermo Fisher Scientific, Yokohama, Japan) coupled to a hybrid quadrupole-Orbitrap mass spectrometer (Thermo Scientific Q Exactive Focus) with heated electrospray ionization (HESI-II). The sample separation was achieved on an Acclaim 120 C18 (2.1 mm × 50 mm, 3 µm, Thermo Scientific, Sunnyvale, CA) column with a flow rate of 0.3 mL/min and a column temperature of 30 °C. Mobile phase A was 10 mM ammonium formate, and mobile phase B was acetonitrile. The following linear gradient program was used for separation, with a total run time of 15 min. The percentage of B solvent (acetonitrile) changed as follows: 0 min, 10%; 1–10 min, 10–90%; 12–12.5 min, 90–10%; 12.5–15 min, 10%. For the measurements, the injection volume was 5 µL. The electrospray ionization (ESI) source was set to a heater temperature of 400 °C, and the sheath gas and auxiliary gas pressures were set to 35 and 10 arbitrary units, respectively. The ion spray voltage was set to 3.5 kV, with a capillary temperature of 320 °C, and the S-lens radio frequency level was 70. Data were acquired by polarity switching in full MS mode.

### 2,4-heptadienal induced 1,N^2^-etheno-ipG (ε−ipG) formation in the presence of tBuOOH

A mixture of ipG (3 mg, 9 µmol), HDE (90%, 16.7 µL, 120 µmol), tBuOOH (70%, 2 µL, 15 µmol), acetonitrile (300 µL), and 100 mM phosphate buffer (pH 7.4, 900 µL) in a capped Eppendorf tube was incubated at 50 °C [17] with occasional mixing to make an emulsion. After 18 h of reaction, 20 µL of the mixture were injected into the HPLC column at conditions: Temperature: 40 °C, Elution, a linear gradient of ethanol concentration in 10 mM ammonium formate, 0–60 min, 15–50%; 60–80 min, 50% (Method 1).

### Isolation of precursors for the ε-ipG formation

Mixture of HDE (90%, 16.7 µL, 120 µmol), tBuOOH (70%, 2 µL, 15 µmol), acetonitrile (300 µL), and 100 mM phosphate buffer (pH 7.4, 900 µL) in a capped Eppendorf tube was incubated at 50 °C with occasional mixing to make an emulsion. After 36 h, 100 µL of the mixture was injected into the HPLC column. Conditions were basically the same as above, except for the elution condition, a linear gradient of ethanol concentration in water, 0–60 min, 15–50%; 60–80 min, 50% (Method 2).

The measurement conditions for mass spectrometry were the same as those for the ipG-adducts, except for the conditions below. Mobile phase A was water, and mobile phase B was methanol. Data were acquired in parallel reaction monitoring (PRM) mode.

For assay of ε-ipG formation, 100 µL of each fraction was mixed with ipG solution (250 μg ipG, 25 μL acetonitrile, 75 μL 100 mM phosphate buffer, pH 7.4), either at 50 °C or 37 °C with tBuOOH (70%, 2μL). After 20 h, 50 μL of each mixture was injected into the HPLC column (Column, Shiseido Capcell Pak C-18, 4.6 × 150 mm, 3 µm), Elution, linear gradient of ethanol concentration in 10 mM ammonium formate, 0–30 min, 15–50% (Method 3).

### Formation of 6-hydroxy- and 6-oxo-2,4-decadienal by auto-oxidation of 2,4-decadienal

A mixture of 2,4-DDE (23.2 μL, 120 μmol), acetonitrile (300 μL), and 100 mM phosphate buffer (900 μL, pH 7.4) in a capped Eppendorf tube was incubated at 37 °C for 20 h with occasional mixing. An aliquot (20 μL) of the mixture was injected into an HPLC column (150 mm). Elution, method 1.

### Effect of pH and oxidants on 6-hydroxy- and 6-oxo-2,4-heptadienal formation

To determine the effect of pH, a mixture of 2,4-HDE (90%, 16.7 μL, 120 μmol), water (855 μL), and 45 μL of 2 M phosphate buffer (pH 7.4), 2 M acetate buffer (pH 4.5) or 0.02N HCl (to make final pH 3.0) in a capped Eppendorf tube was incubated at 37 ºC for 103 h with occasional mixing. An aliquot of the mixture was injected into an HPLC column (150 mm) at appropriate time points. Elution, method 1.

For the effect of oxidants, a mixture of HDE (90%, 16.7 μL, 120 μmol), acetonitrile (300 μL), 100 mM phosphate buffer (pH 7.4, 900 μL), in the presence or absence of tBuOOH (70%, 2 μL, 15 μmol), or H_2_O_2_ (30%, 1.5 μL, 15 μmol) in a capped Eppendorf tube was incubated at 37 °C for 100 h with occasional mixing to make an emulsion. An aliquot of the mixture was injected into an HPLC column (150 mm) at appropriate time points. Elution, method 1.

### Hemin plus tBuOOH stimulate 6-hydroxy- and 6-oxo-2,4-heptadienal formation

Hemin was dissolved in 20 mM NaOH (2,17 mg/mL). A mixture of HDE (4.2 μL), acetonitrile (75 μL), water (214 μL), 2 M Buffer (pH 4.5 or 7.4, 11.3 μL), in the presence or absence of hemin (5.5 μL), or tBuOOH (1 μL), was incubated at 37 ºC for 5 h, with occasional mixing. An aliquot of the mixture was injected into an HPLC column (150 mm) at appropriate time points. Elution, method 1.

The measurement conditions for the mass spectrometry of product peak 3 were the same as those for ipG-adducts, except that the mobile phase was changed. Mobile phase A was water, and mobile phase B methanol. The injection volume was 10 µL.

### MnO_2_ oxidation of compound 2 (6-OH-HDE)

An excess amount of MnO_2_ (8.8 mg) was added to a solution of compound 2 (6-hydroxy-HDE) (69 μg) in acetone (0.5 mL) and reacted at 22 °C for 1 h. The conversion of 2 to 1 was confirmed by HPLC analysis (retention time, UV spectrum) and mass spectrum.

### Detection of 6-hydroxy- and 6-oxo-2,4-alkadienal in heated cooking oil

The heated oil (500 μL) and ethanol (500 μL) were vigorously mixed, and the upper phase was collected (400 μL) after centrifugation. The ethanol extraction was repeated twice. The combined extract was evaporated to dryness under reduced pressure, the initial buffer for HPLC (100 μL) was added to make an emulsion, and an aliquot (50 μL) was injected into an HPLC column (Shiseido, 100 mm, 3 µm). Elution, method 3.

The mass spectrometry determination of isolated peaks by the above HPLC was the same as “*Isolation of precursors for the ε-ipG formation”* except for changing CE:20 to CE:10. The injection volume was 10 µL. The mass chromatogram is represented as the result of selected ion monitoring (SIM) based on the molecular ion of each compound.

### Effect of tBuOOH for the formation of 1,N^2^-etheno-ipG by 6-oxo- and 6-hydroxy-2,4-alkadienal

A mixture of ipG (375 nmol), HDE-1 (10.7 nmol), or HDE-2 (45.5 nmol), acetonitrile (25 μL), and 100 mM phosphate buffer (pH 7.4, 75μL) was incubated at 37 °C for 95 h, in the presence or absence of tBuOOH (7.5 μmole). An aliquot of the mixture was injected into an HPLC column (150 mm). Elution, method 3.

### Comparison of ε-ipG formation from ipG between HDE-1, HDE-2, and HDE

A mixture of ipG (375 nmol), HDE-1 (20 nmol), HDE-2 (20 nmol), or HDE (20 nmol), tBuOOH (7.5 μmol), acetonitrile (25 μL), and 100 mM phosphate buffer (pH 7.4, 75 μL) was incubated at 37 °C for 81 h. An aliquot of the mixture was injected into the HPLC column (150 mm) at appropriate time points. Elution, method 3.

### Formation of εGua in DNA

DNA was dissolved in distilled water at 1 mg/mL. A part of the solution was heated in boiling water for 15 min and rapidly cooled in ice water to make single-strand-DNA (ss-DNA).

For 6-oxo-compounds, a mixture of DNA (95 μL), 400 mM phosphate buffer (pH 7.4, 5 μL), HDE-1 (21.4 nmol), or DDE-1 (21.4 nmol) dissolved in acetonitrile (25 μL), and water (75 μL), was incubated at 37 °C for 20 h, in the presence of tBuOOH (15 μmol). For 6-OH-compounds, a mixture of DNA (95 μL), 400 mM phosphate buffer (pH7.4) (5 μL), HDE-2 (91 nmol), or DDE-2 (91 nmol), acetonitrile (25 μL), water (75 μL) was incubated at 37 °C for 20 h, in the presence of tBuOOH (15 μmol). DNA was precipitated by adding 2 M NaOAc (35 μL) and ethanol (470 μL) and cooling at –20 ºC. DNA was collected by centrifugation and washed with ethanol (300 μL). Dried DNA was mixed with 0.1N HCl (160 μL) and incubated at 37 °C for 15 h. The solution was neutralized to pH 6 with 0.2 M sodium carbonate-bicarbonate buffer (pH 9.6, 40 μL).

For the liquid chromatography-tandem mass spectrometry (LC-MS/MS) measurement of εGua in DNA, the sample separation was achieved using L-column 3 C18 (2.1 mm × 100 mm, 3 µm, CERI, Tokyo, Japan) with a flow rate of 0.3 mL/min and a column temperature of 30 °C. Mobile phase A was 10 mM ammonium formate and mobile phase B acetonitrile. The following linear gradient program was used for separation, with a total run time of 20 min. The percentage of B solvent (acetonitrile) changed as follows: 0 min, 1%; 8–13 min, 1–10%; 13–13.5 min, 10–80%; 14.5–15 min, 80–1%. The sample injection volume was 5 µL. The HESI source condition was the same as the ipG adduct measurement. The data were acquired in the PRM mode. In this mode, a single precursor ion, [M + H]^+^ (m/z 176.05669), was selected. After fragmentation in the HCD cell at CE 30, the resulting MS/MS product ions were detected at a resolution of 70,000. The most abundant ion (m/z 121.0511) was used for quantification.

### Decomposition of HDE-1, HDE-2, and 4,5-epoxy-2-heptenal in a gastric condition

Solutions of HDE-1, HDE-2, and 4,5-epoxy-2-heptenal (48 μL each) were mixed with glacial acetic acid (3 μL) to make a pH 3 solution and incubated at 37 °C for 8.5 h. An aliquot of the solution was injected into the HPLC column (150 mm) at appropriate time points. Elution, 30% aqueous ethanol.

## Results

### Analysis of ipG-adducts produced by heated cooking oil

**Fig. 1:**
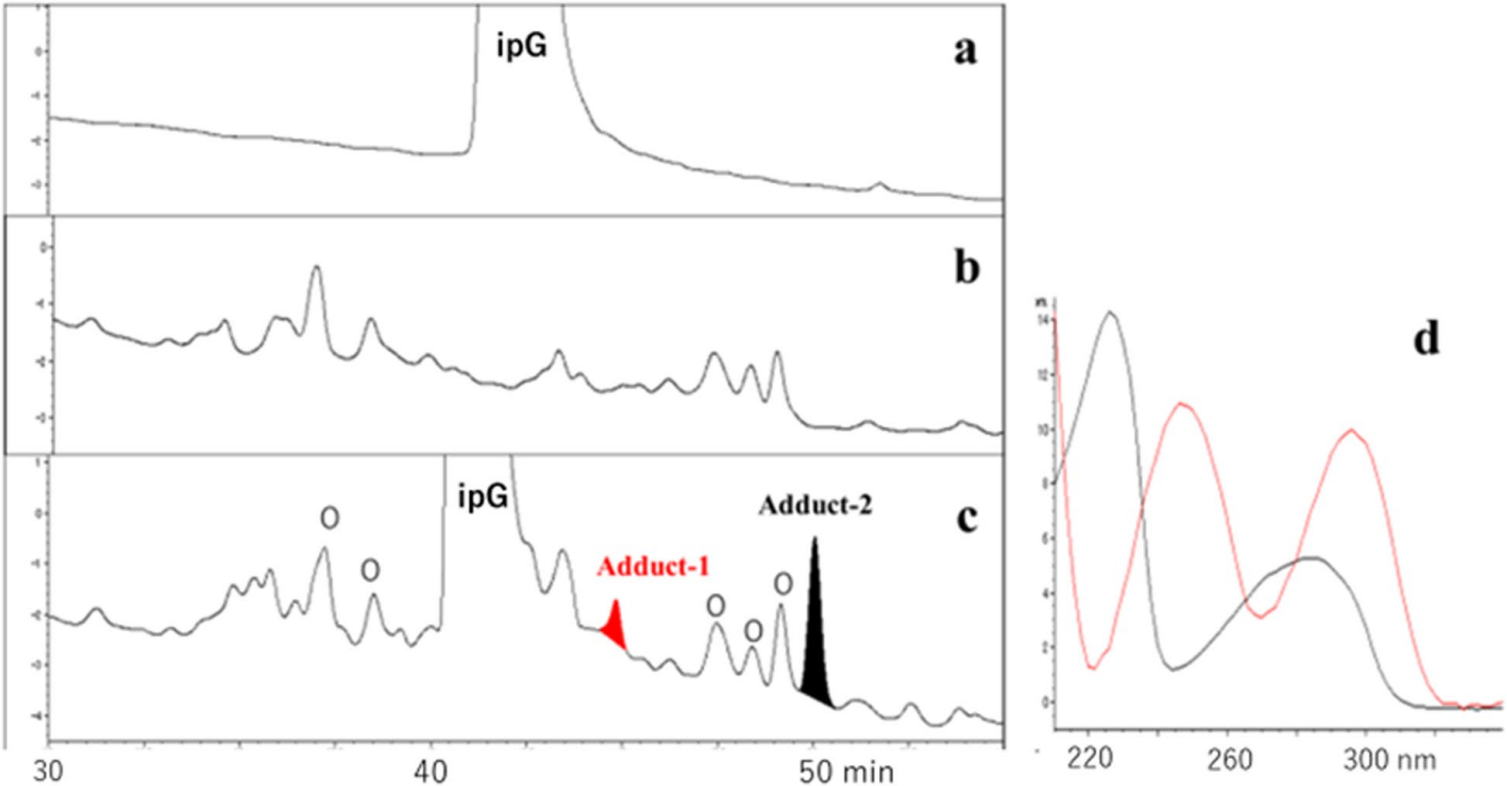
Analysis of adducts formed by the reaction of ipG and heated cooking oil. **a** ipG control, **b** heated oil control, **c** reaction of ipG and heated cooking oil, **d** UV spectra of adduct-1(red) and adduct-2 (black). Heated oil components were shown by “o”

In the reaction mixture of ipG and heated oil, adduct-1 and -2, which were absent in the ipG- and heated oil-controls (Fig. 1a and b), were detected by HPLC (Fig. 1c). They exhibited characteristic UV spectra (Fig. 1d). No other adducts were detected after prolonged gradient elution. The structures of adduct-1 (minor) and adduct-2 (major) were deduced to be 8-OH-ipG and 1,N^2^-etheno-ipG ( ε − ipG), respectively, by MS analysis (adduct-1: *m/z* 340.1254[M + H]^+^, 338.1108[M-H]^−^; adduct-2: *m/z* 348.1304[M + H]^+^, 346.1158[M-H]^−^), UV spectra, and further confirmed by comparing the synthetic samples (retention time in HPLC, UV- and mass spectra).

### 2,4-heptadienal induced 1,N^2^-etheno-ipG (ε−ipG) formation in the presence of tBuOOH

**Fig. 2:**
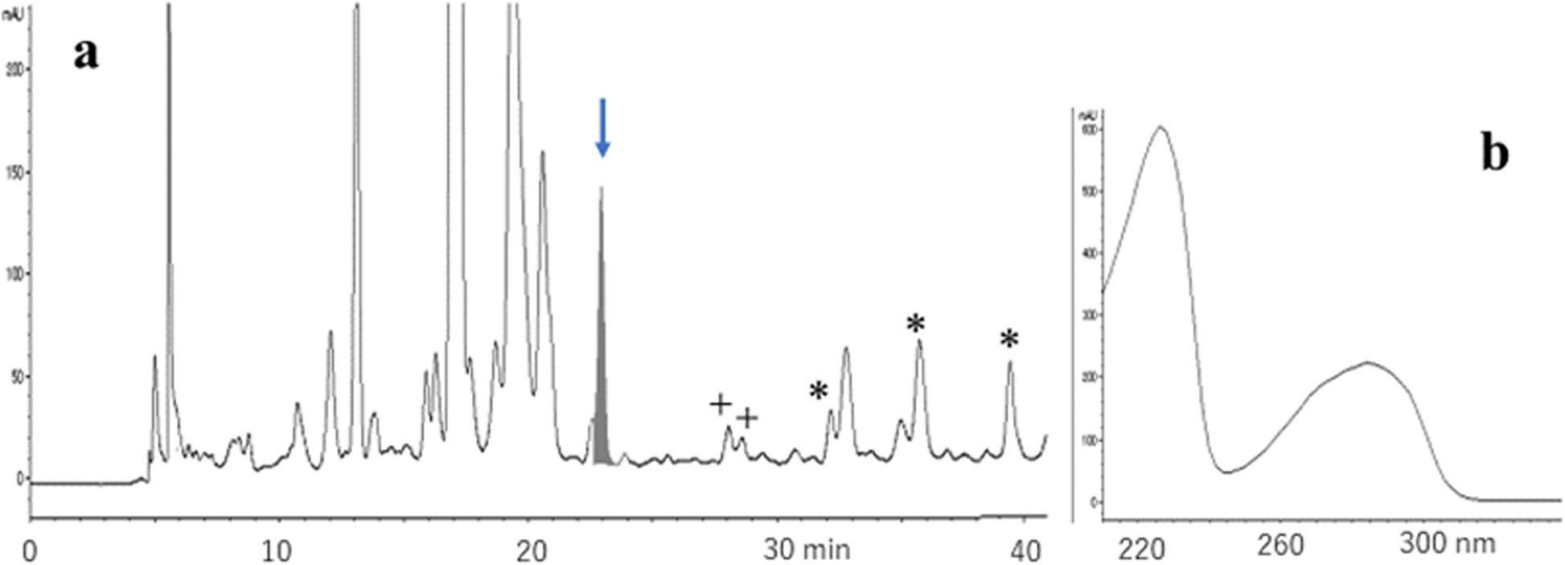
Analysis of a reaction mixture of ipG, 2,4-heptadienal, and tBuOOH. **a** Chromatogram at 280 nm, **b** UV spectrum of the peak indicated by an arrow. + 1,N^2^-etheno-type adduct, * 1,N^2^-ethano-type adduct, deduced from UV spectra

Loureiro et al. reported the reaction of 2’-deoxyguanosine (dG) with 2,4-decadienal and tBuOOH [17]. In the present study, the formation of ε − ipG was confirmed by the reaction of ipG with the analogous lipid peroxidation product 2,4-heptadienal (HDE), which has been detected in fried foods in comparable amounts to DDE [18]. As shown in Fig. 2a, the UV spectra of ε − ipG (major, indicated by arrow) and other minor adducts similar to those of etheno- and ethano-type adducts with side chains were detected. In the presence of H_2_O_2_, instead of tBuOOH, a similar adduct profile was observed (data not shown). The yield of ε − ipG in the presence of tBuOOH or H_2_O_2_ was 5- to 6-fold higher than that in the absence of oxidants, suggesting that the oxidation steps of HDE are important for ε − ipG formation. We deemed tBuOOH to be more important than H_2_O_2_ for model reactions, because large amounts of alkyl hydroperoxides are present in heated cooking oil. Therefore, tBuOOH was mainly used as an oxidant in the present study.

### Identification of precursors for the ε-ipG formation in oxidized HDE and DDE

**Fig. 3:**
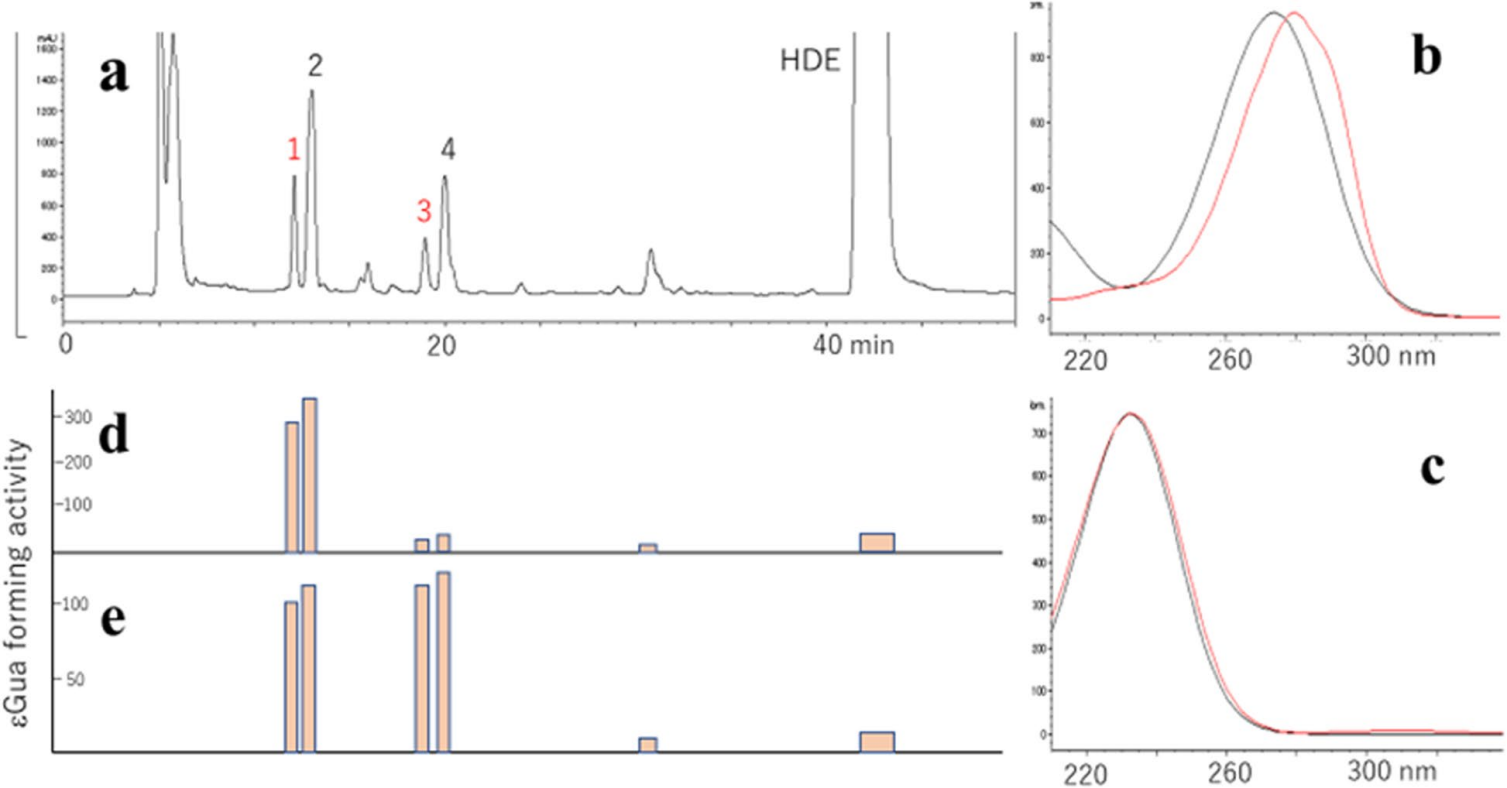
Isolation of precursors for the ε-ipG formation. **a** Chromatogram at 280 nm (bandwidth, 100 nm). **b** UV spectra of peak 1 (red) and 2 (black). **c** UV spectra of peak 3 (red) and 4 (black). **d** εGua forming activity at 50 °C. **e** εGua forming activity at 37 °C with tBuOOH. The bars show the peak area of formed ε-ipG

**Fig. 4:**
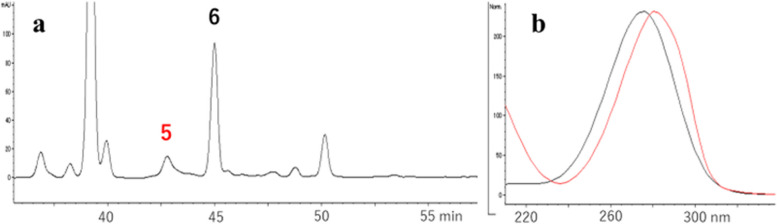
Detection of DDE oxidation products. **a** Chromatogram at 280 nm, **b** UV spectra of peak 5 (red) and peak 6 (black)

To identify the causative agents of ε* − *ipG formation, HDE was incubated with tBuOOH, fractionated by HPLC (Fig. 3a), and each fraction was assayed for ε* − *ipG formation from ipG. Under assay conditions of 50 °C, pH 7.4, without oxidants, compounds 1 (HDE-1) and 2 (HDE-2) were positive (Fig. 3d), while under the assay conditions of 37 °C, pH 7.4, with tBuOOH, compounds 1, 2, 3, and 4 were positive (Fig. 3e). Based on the UV spectra (Fig. 3b), compound 2 should retain two conjugated double bonds, whereas compound 1 has a characteristic UV spectrum with a shoulder, similar to that of muconaldehyde (hexa-2,4-dienedial) [19]. Compounds 3 and 4 showed UV spectra similar to that of a known lipid peroxidation product, 4,5-epoxy-2-decenal (Fig. 3c) [20]. They were supposed to be two stereoisomers. The structure was confirmed by mass spectrometry (3, M-H = 125.0608, C_7_H_9_O_2_; 4, M-H = 125.0609, C_7_H_9_O_2_). By auto-oxidation of 2,4-decadienal (DDE), the formation of analogous products 5 and 6 (DDE-1 and DDE-2) with similar UV spectra to those of 1 and 2 (HDE-1 and HDE-2) were also observed (Fig. 4). Further studies were focused on new compounds, 1, 2, 5 and 6.

**Fig. 5:**
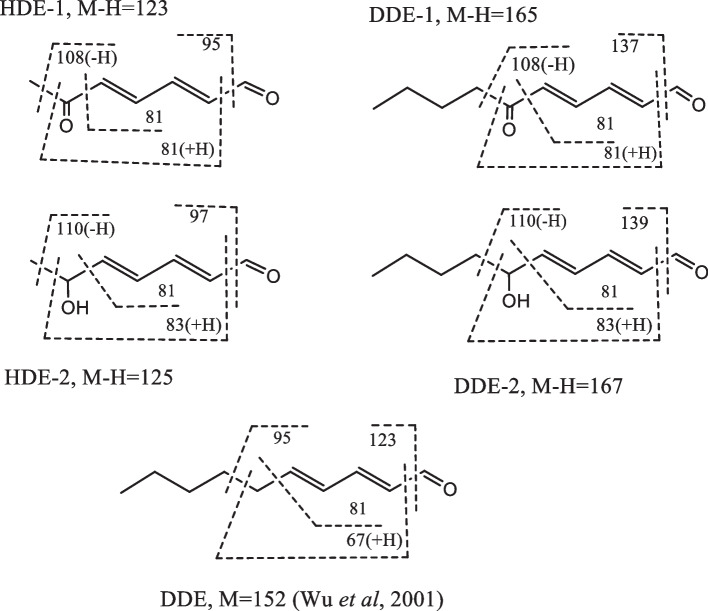
Structures and mass spectral data of HDE-1, HDE-2, DDE-1, and DDE-2

Since the yields of oxidation products were considerably low (less than 1%), and isolation of enough amount of these compounds for NMR measurement was challenging, we identified unknown compounds primarily using MS fragmentation. Based on MS spectral data, the structures of compounds, 1, 2, 5, and 6 were deduced to be 6-oxo-, 6-hydroxy-2,4-HDE and 6-oxo-, 6-hydroxy-2,4-DDE, respectively. Data on molecular ions (M-H) and fragmentation patterns are shown in Fig. 5. For comparison, the fragmentation pattern of DDE [21] is shown. These structures were also confirmed by high-resolution MS spectra (1: *m/z* 125.0608[M-H]^−^, C_7_H_9_O_2_; 2: *m/z* 123.0452[M-H]^−^, C_7_H_7_O_2_; 5: *m/z* 167.1078[M-H]^−^, C_10_H_15_O_2_; 6: *m/z* 165.0921[M-H]^−^, C_10_H_13_O_2_) (S1). The structures of 1 and 2 were also compatible with the fact that 2 was quantitatively converted to 1 by MnO_2_ oxidation and mechanistic consideration that 1 (6-oxo-derivative) and 2 (6-hydroxy-derivative) were formed from 6-OOH-derivative, as shown below.

### Effect of oxidants, hemin and pH on the formation of oxidized products from HDE

**Fig. 6:**
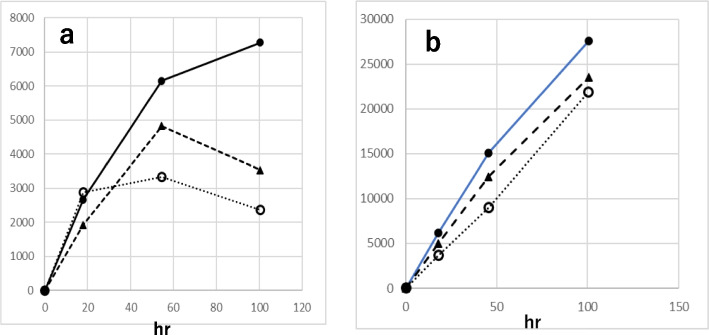
Effect of oxidants on the formation of oxidized products from HDE. **a** Formation of HDE-1 and **b** formation of HDE-2. (●) without oxidant, (▲) with H_2_O_2_, and (○) with tBuOOH

**Fig. 7:**
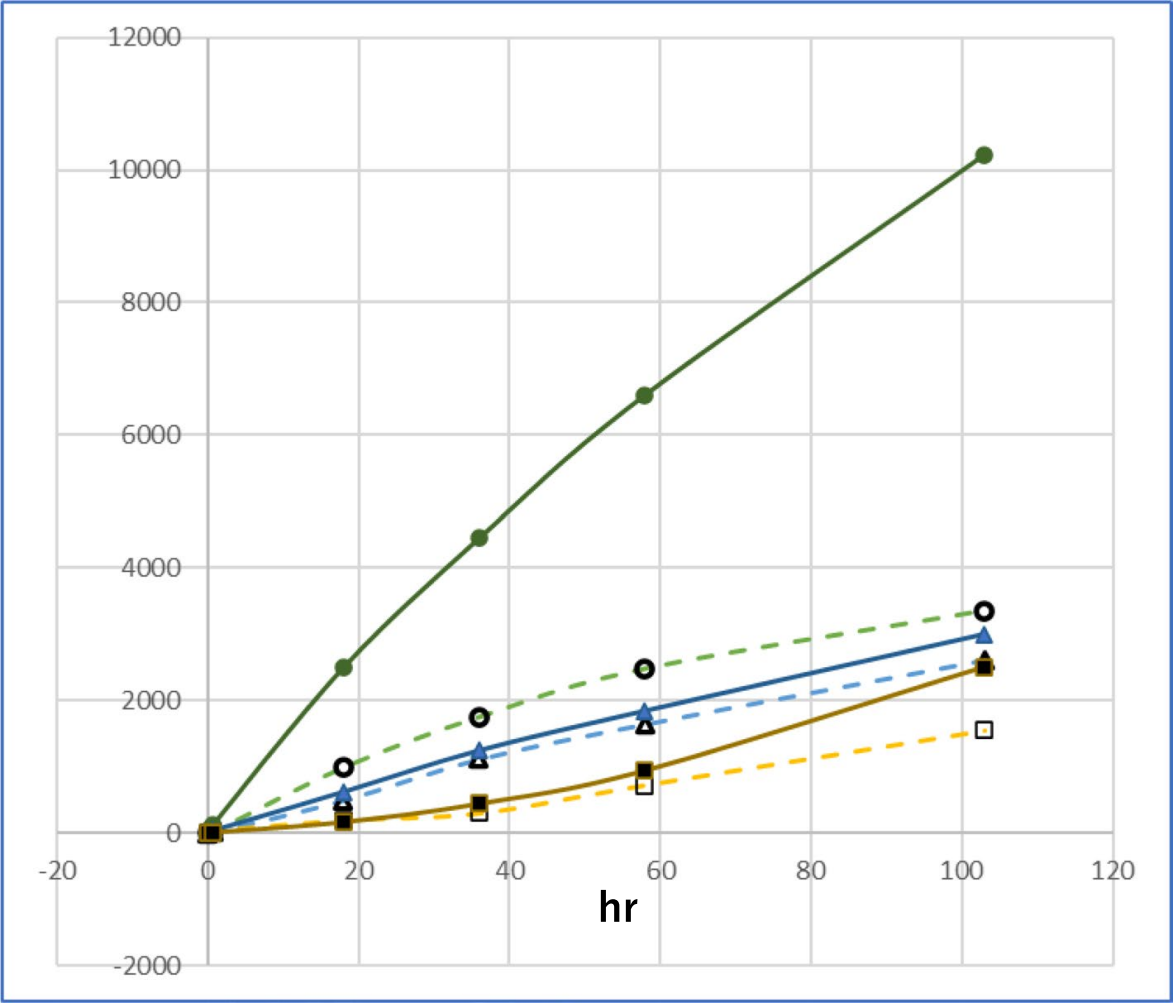
Effect of pH on the formation of oxidized products from HDE. (●) HDE-2 at pH 7.4, (○) HDE-1 at pH 7.4, (▲) HDE-2 at pH 4.5, (△) HDE-1 at pH 4.5, (■) HDE-2 at pH 3.0, (□) HDE-1 at pH 3.0

**Fig. 8:**
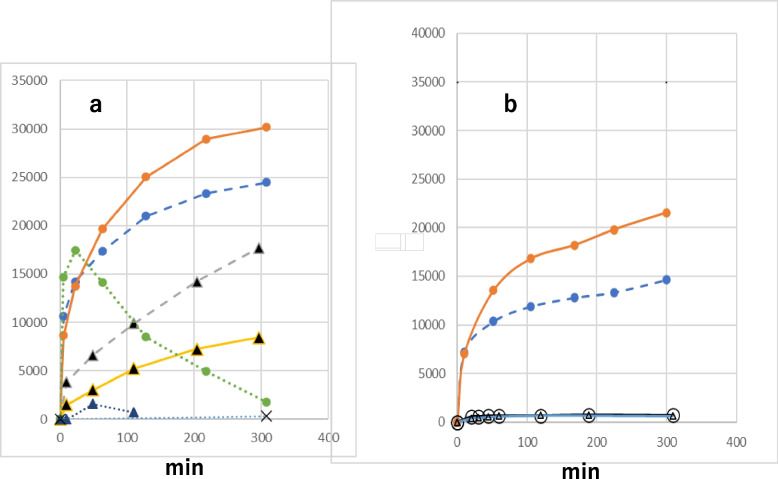
Effect of hemin and tBuOOH on HDE oxidation. **a** pH 4.5, (●―●) formation of HDE-2 with hemin, tBuOOH, (●---●) formation of HDE-1 with hemin, tBuOOH, (●…●) formation of peak 3 of Fig. 9a with hemin, tBuOOH, (▲---▲) formation of HDE-1 with hemin, (▲―▲) formation of HDE-2 with hemin, (▲…▲) formation of peak 3 of Fig. 9a with hemin. (x) without hemin and tBuOOH (HDE-1, HDE-2). **b** pH 7.4, (●―●) formation of HDE-2 with hemin, tBuOOH, (●---●) formation of HDE-1 with hemin, tBuOOH, (○―○) formation of HDE-1 with hemin, (△―△) formation of HDE-2 with hemin

**Fig. 9:**
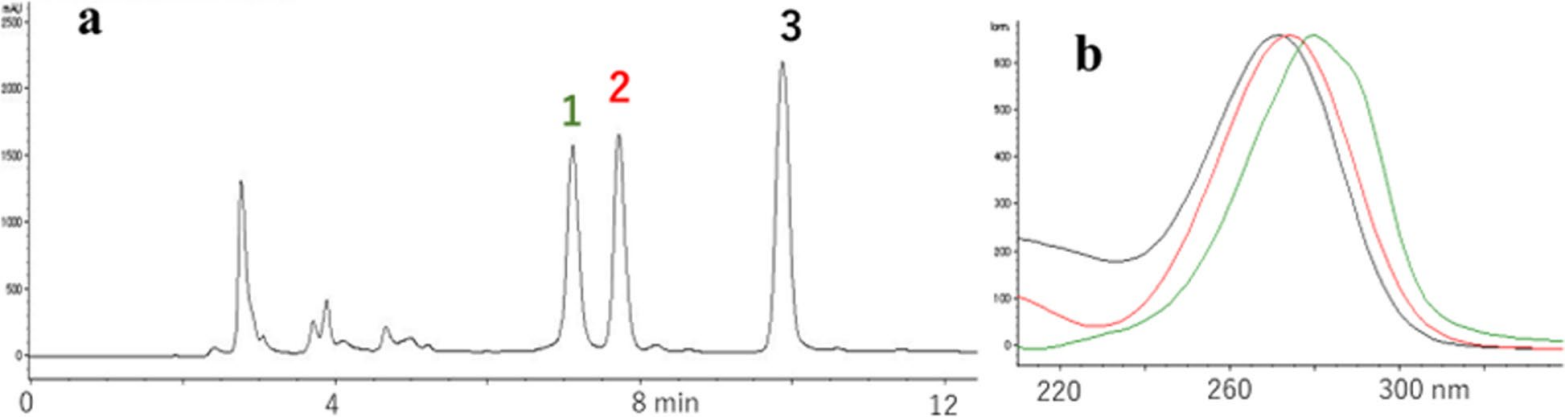
Analysis of a reaction mixture of HDE, hemin, and tBuOOH at 23 min of incubation time. **a** Chromatogram, **b** UV spectra of 1 (green), 2 (red) and 3 (black)

**Fig. 10:**
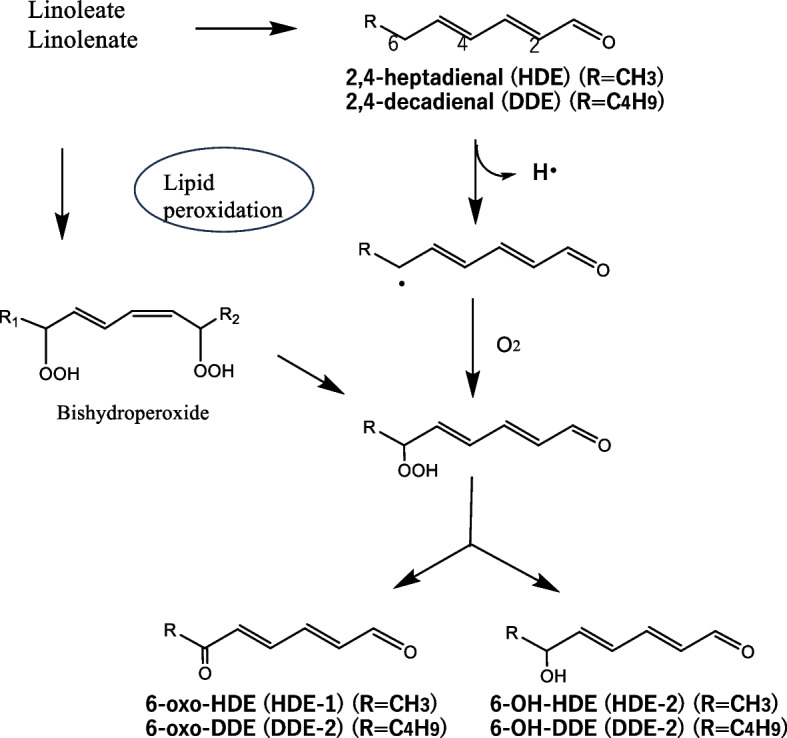
Scheme for 6-hydroxy and 6-oxo-2,4-alkadienal formation

The yield of HDE-1 and -2 from HDE was in the order, with no oxidant > with H_2_O_2_ ~ with tBuOOH (Fig. 6), while the yields of compounds 3 and 4 (4,5-epoxy-2-heptenal) were 5 to 6 times higher with H_2_O_2_ or tBuOOH than without oxidant (data not shown). The latter is compatible with the fact that epoxidation of the double bond occurs with oxidants H_2_O_2_ and tBuOOH. Regarding the effect of pH, the yields of HDE-1 and -2 from HDE were in the order of pH 7.4 > pH 4.5 > pH3.0 (Fig. 7). The combination of hemin and tBuOOH strongly stimulated the formation of HDE-1 and-2. (Fig. 8). The yield was higher at pH 4.5 than pH 7.4. In the early period (23 min) of the hemin plus tBuOOH reaction at pH 4.5, in addition to HDE-1 and -2, the third product (peak 3) appeared in HPLC (Fig. 9a), which was supposed to be a 6-OOH derivative deduced from MS analysis (*m/z* 141.0557[M-H]-, C_7_H_9_O_3_). It decreased with time with the concomitant formation of HDE-1 and -2 (Fig. 8a). From these data, the right pathway in Fig. 10 was proposed for 6-hydroxy- and 6-oxo-2,4-alkadienal formation.

### Detection of 6-hydroxy- and 6-oxo-2,4-alkadienal in heated cooking oil

**Fig. 11:**
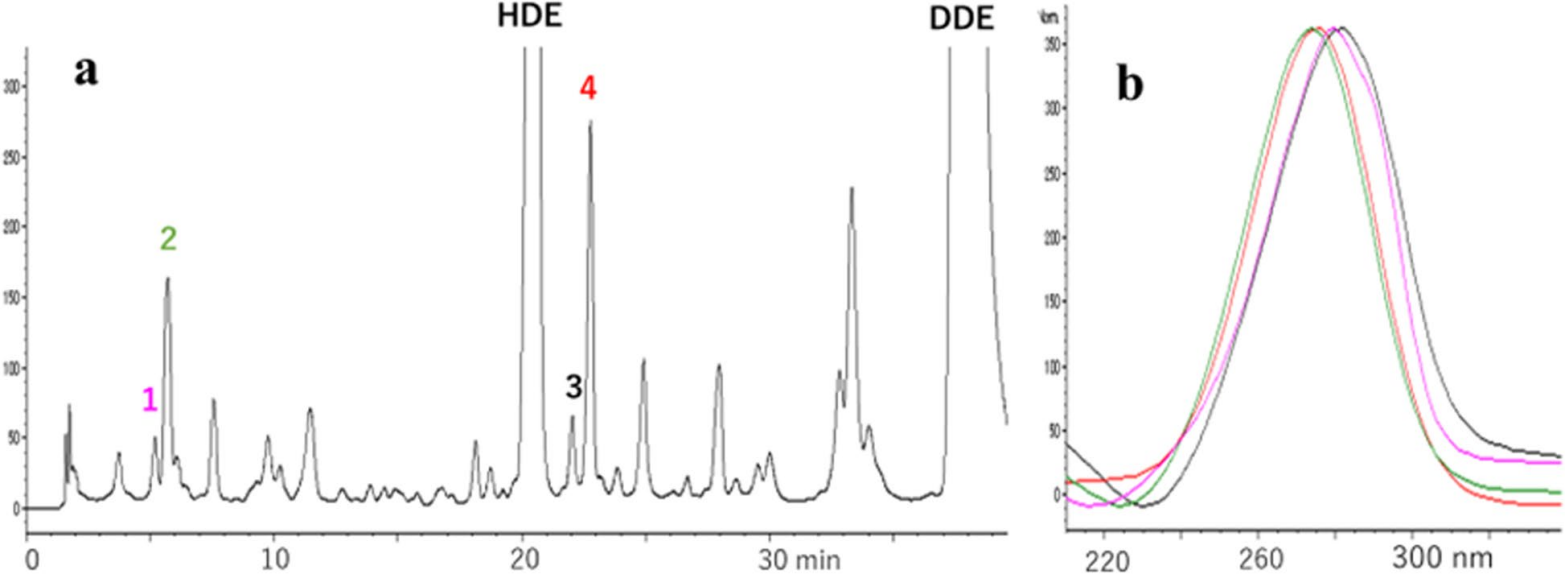
Detection of 6-hydroxy- and 6-oxo-2,4-alkadienal in heated cooking oil. **a** Chromatogram, **b** UV spectra of 1 (pink), 2 (green), 3 (black) and 4 (red)

**Fig. 12:**
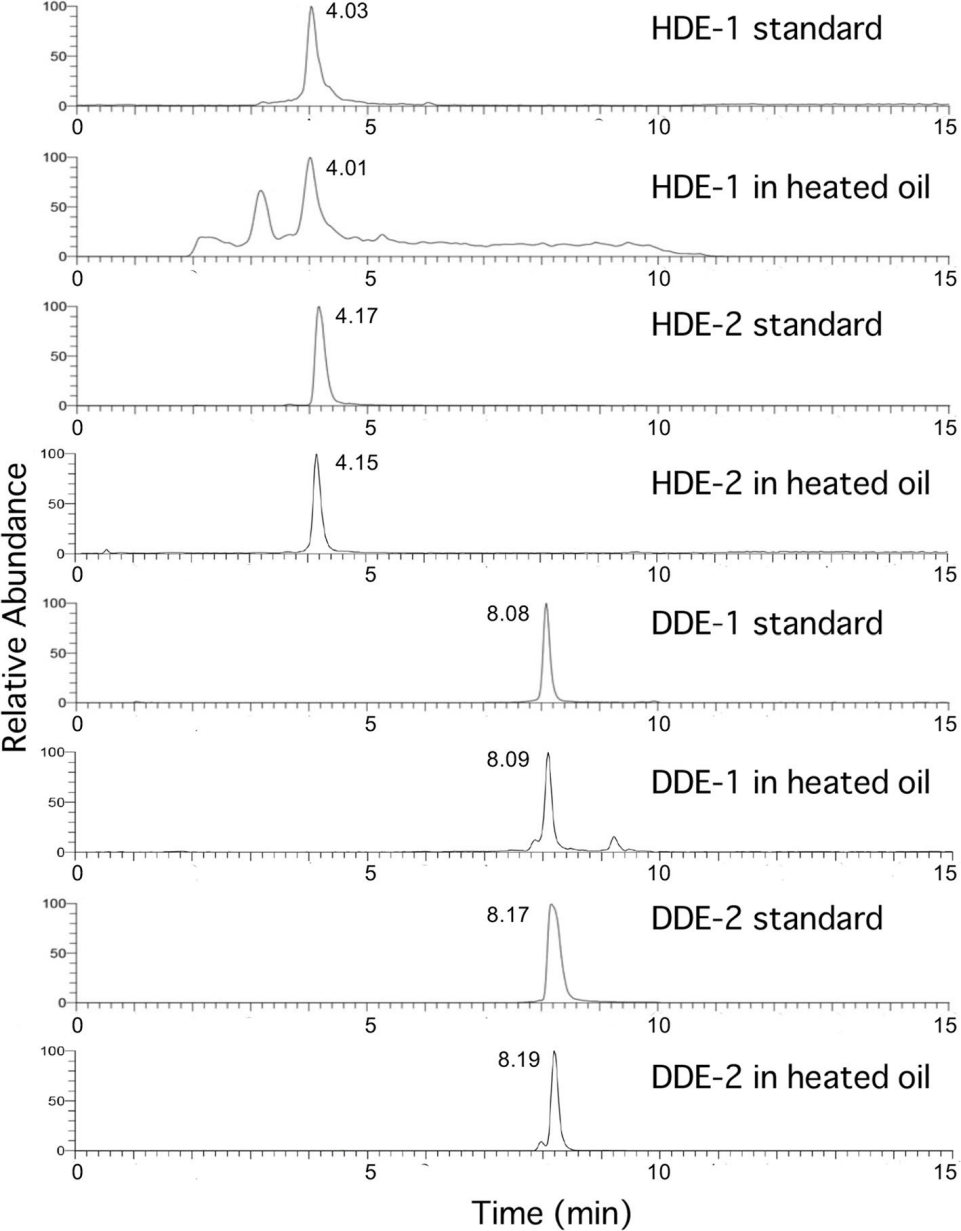
Comparison of 6-hydroxy- and 6-oxo-2,4-alkadienals standards and those detected in heated cooking oil. Four standards and fractions 1–4 obtained by fractionation of the heated oil were compared by LC/MS (SIM)

**Fig. 13:**
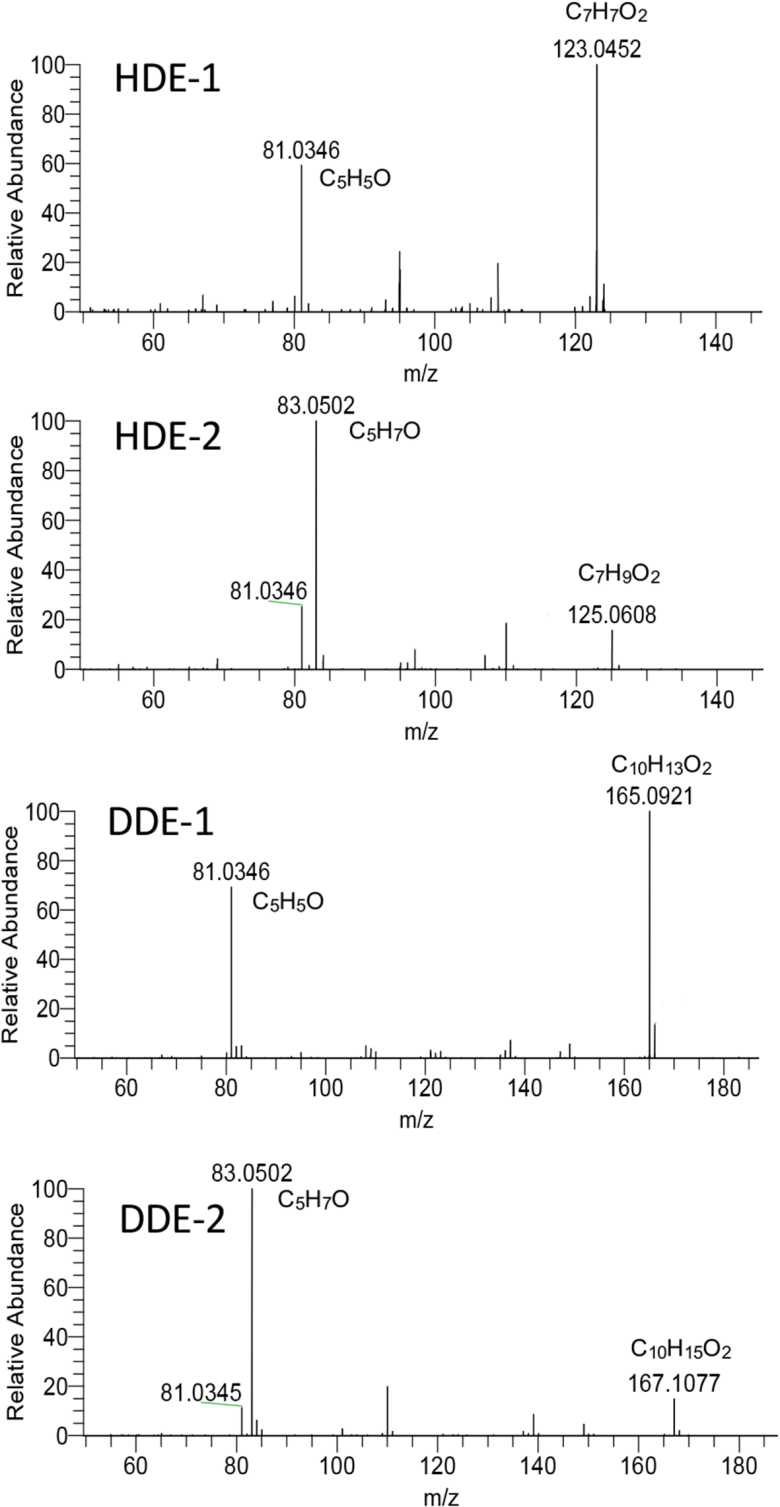
Mass spectra of 6-oxo- and 6-hydroxy-2,4-alkadienals detected in heated cooking oil

Figure 11a shows the HPLC chromatogram of the fractionation of heated oil components. Four peaks appeared at the same elution positions and showed the same UV spectra (Fig. 11b) as those of standards 1–4 (HDE-1, HDE-2, DDE-1, and DDE-2, respectively). None of these peaks were detected before heating. The identity of these compounds was also confirmed by LC-MS analysis [SIM chromatogram (Fig. 12), high-resolution MS (Fig. 13), fragmentations (Fig. 13)]. During the analysis of HDE-1 in heated oil using LC–MS (Fig. 12), two peaks appeared in the SIM chromatogram. However, the reason for the origin of the extra peak is not known. Amounts of 6-hydroxy-2,4-alkadienals (HDE-2 plus DDE-2) in heated oil were estimated to be 3.8–4.6 μg/g. The two large peaks in Fig. 11 were identified as HDE and DDE, based on a comparison of retention times and mass- and UV spectra with those of authentic samples.

### Decomposition of 4,5-epoxy-2-heptenal in a gastric condition

**Fig. 14:**
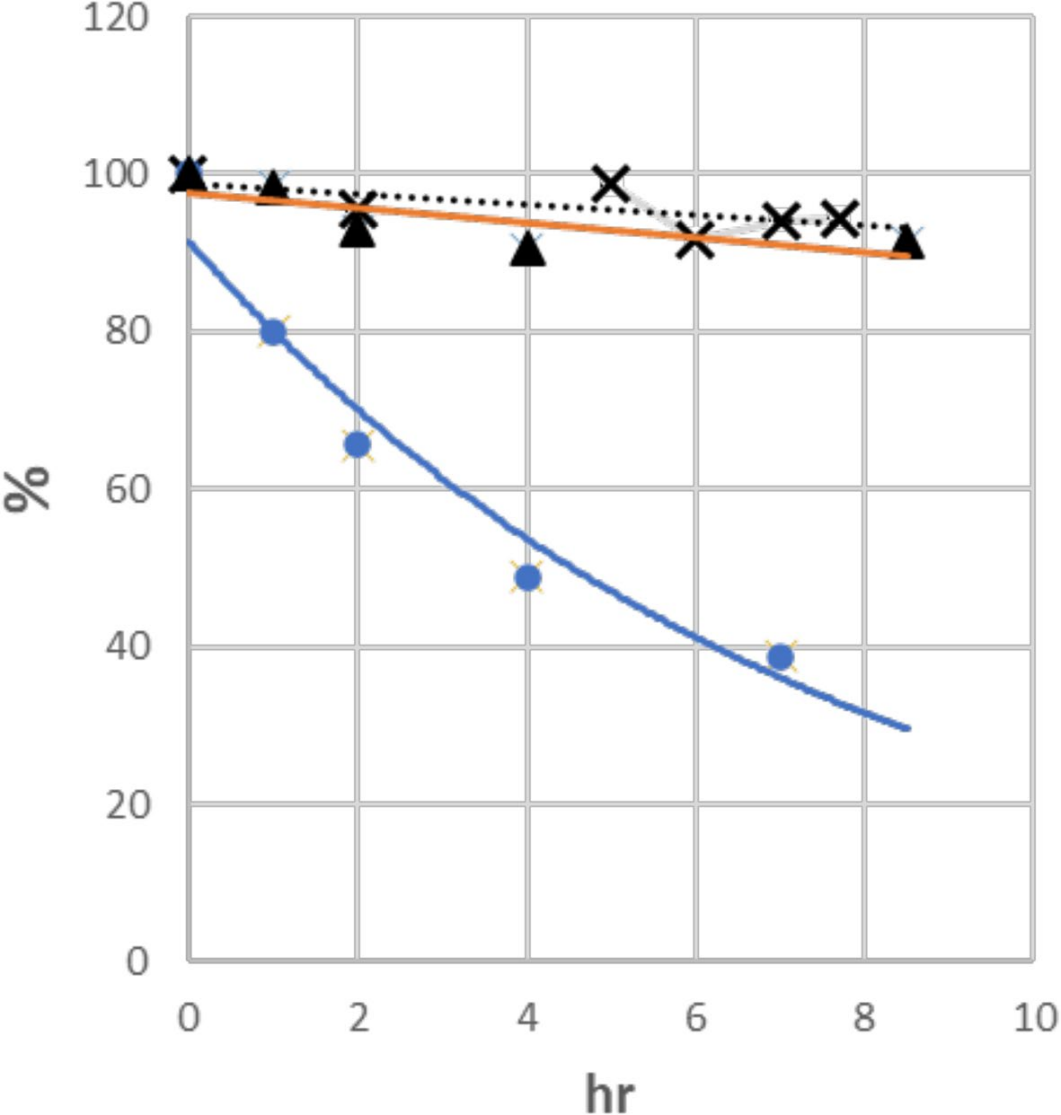
Stability of HDE-1 (x), HDE-2 (▲) and 4,5-epoxy-2-heptenal (●)

In acidic gastric conditions, many epoxy-compounds decompose, including α-allylic epoxide (epoxide adjacent to double bond) [22, 23]. Figure 14 shows time course of decomposition of HDE-1, HDE-2 and 4,5-epoxy2-heptenal, at pH 3, 37 °C. Only 4,5-epoxy2-heptenal showed significant decomposition (half-life, 4 h) at the gastric condition pH 3.

### Effect of tBuOOH for the formation of 1,N^2^-etheno-ipG by 6-oxo- and 6-hydroxy-2,4-alkadienal

**Fig. 15:**
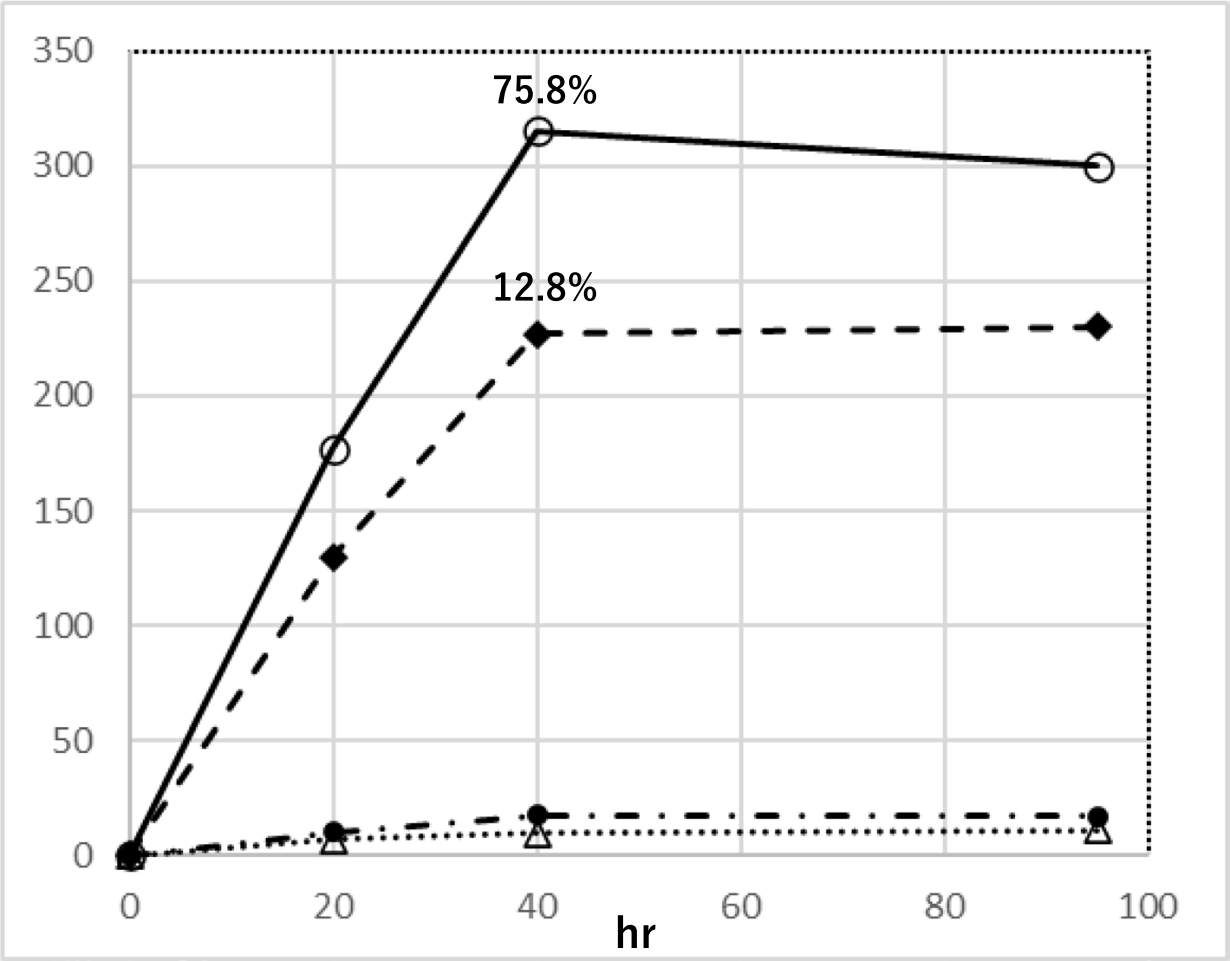
Effect of tBuOOH for the formation of 1,N^2^-etheno-ipG by HDE-1 and HDE-2. (○―○) HDE-1, tBuOOH, (◆---◆) HDE-2, tBuOOH, (●) HDE-1, (△) HDE-2. Peak area (A280nm) of ε-ipG were plotted. The yields (%) of ε-ipG calculated from initial concentrations of HDE-1 and -2 were also shown

**Fig. 16:**
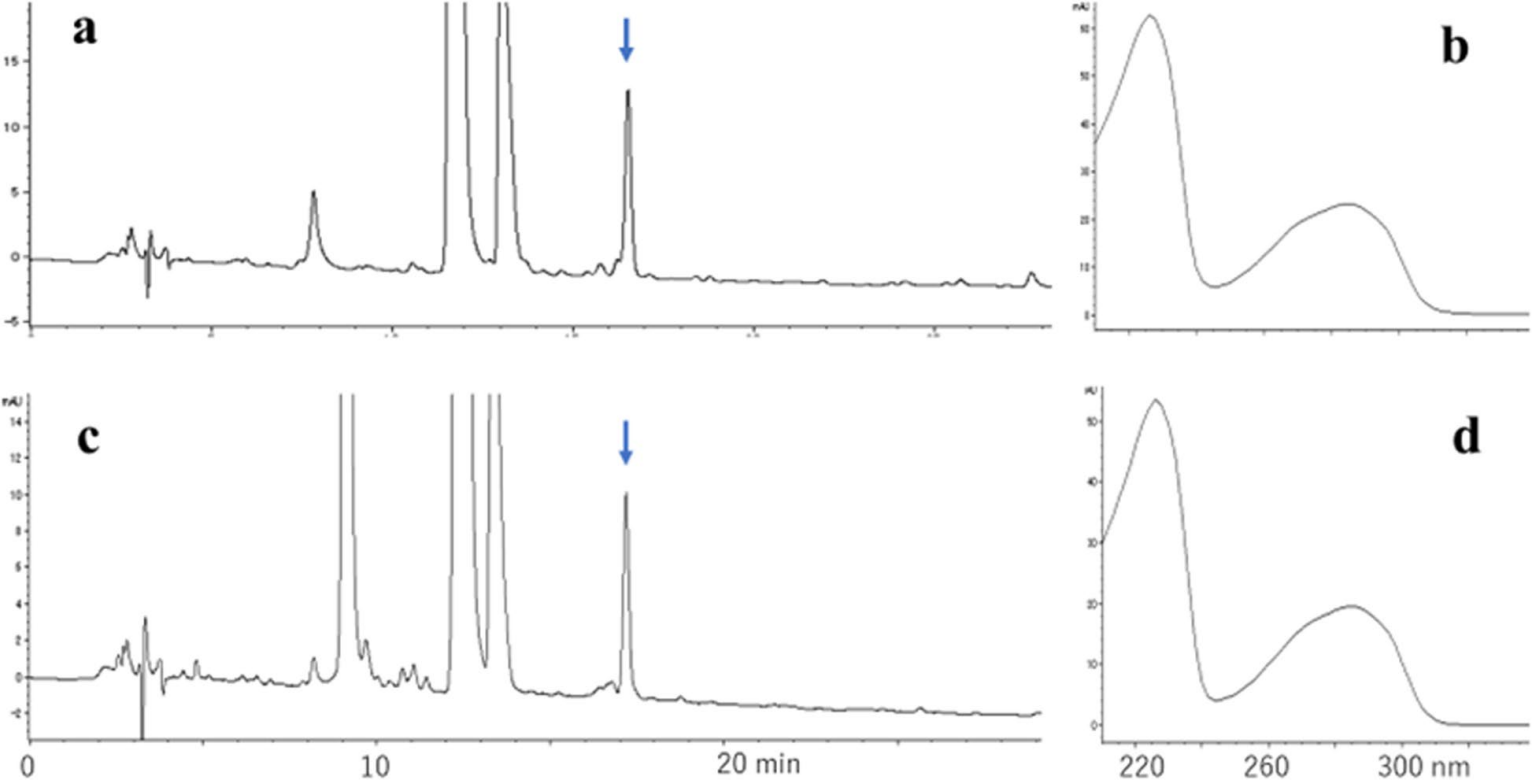
Analysis of reaction products of ipG and HDE-1, or HDE-2, in the presence of tBuOOH. **a** Reaction of ipG, HDE-1 and tBuOOH, **b** UV spectrum of the peak indicated by arrow, **c** reaction of ipG, HDE-2 and tBuOOH, **d** UV spectrum of the peak indicated by arrow

**Fig. 17:**
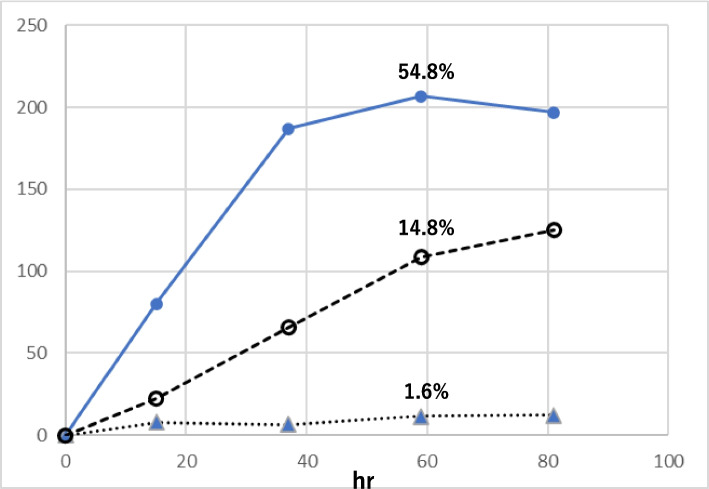
Comparison of 1,N^2^-etheno-ipG formation by HDE-1 (●), HDE-2 (○), and HDE (▲), in the presence of tBuOOH. Peak area (280 nm) of ε-ipG were plotted. The yields (%) of ε-ipG calculated from initial concentrations of HDE-1 and -2 were also shown

In Fig. 2, the data suggest that oxidation steps are required for ε − ipG formation. To confirm this point, yields of ε − ipG from ipG by HDE-1 or HDE-2 in the presence and absence of tBuOOH were compared at 37 °C (Fig. 15). In the presence of tBuOOH, ε − ipG formation from HDE-1 and HDE-2 was dramatically increased (yield, 75.8%, and 12.8%, respectively, calculated from the initial concentrations of HDE-1 and HDE-2), as compared to those without tBuOOH. Therefore, tBuOOH is an important factor in the formation of ε − ipG. The HPLC profile shows that ε − ipG is the only product of these reactions, without any etheno-, ethano-, and propano-type ipG derivatives with side chains (Fig. 16). In a reaction of ipG with HDE-2 in the presence of H_2_O_2_, the yield of ε − ipG was 2 to 3 times lower than that in the presence of tBuOOH. Figure 17 compares the formation of ε − ipG with the same concentration of HDE-1, HDE-2, and HDE, in the presence of tBuOOH. The formation rate was in the order HDE-1 > HDE-2 >  > HDE.

### Formation of εGua in DNA

**Fig. 18:**
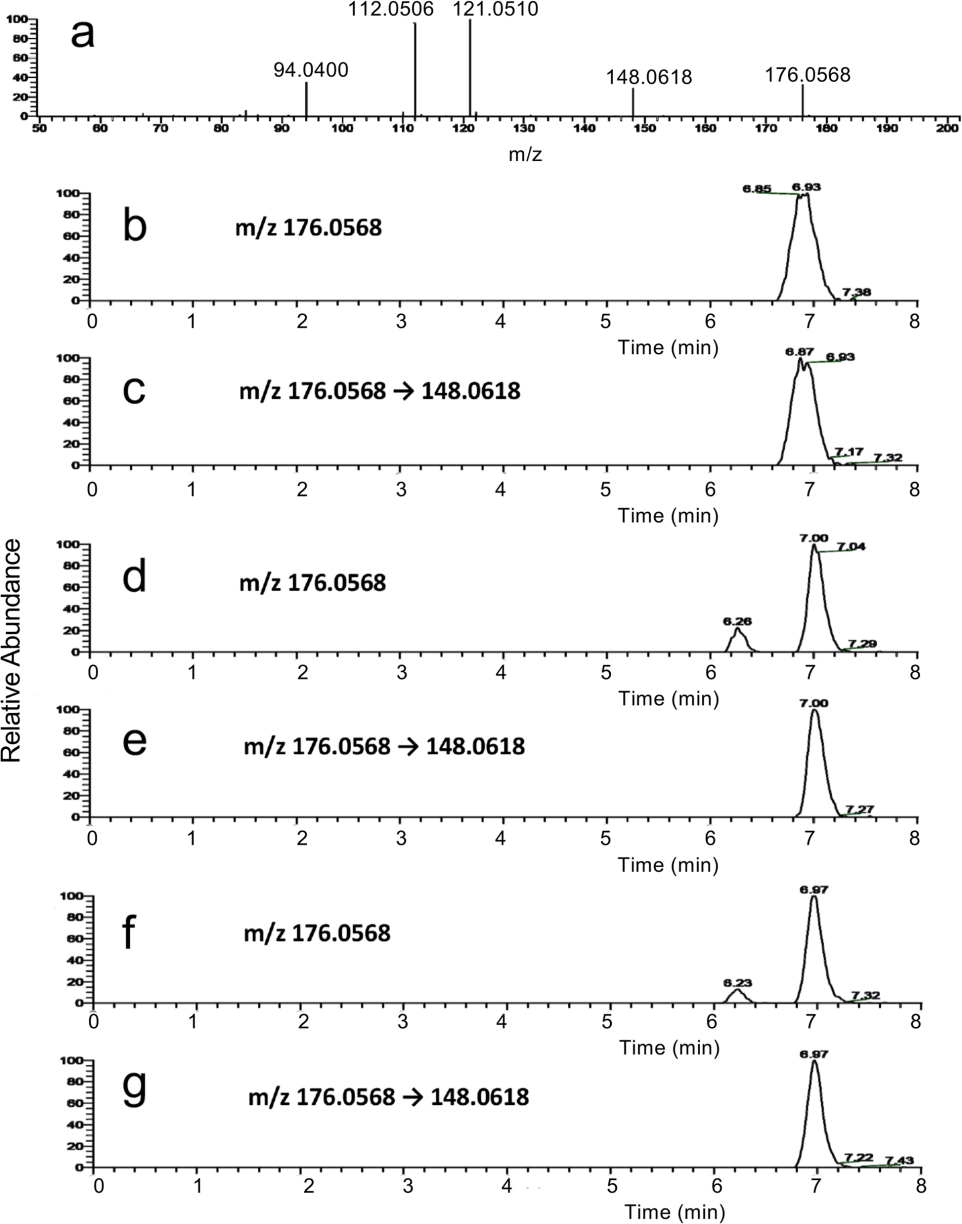
Detection of εGua in DNA treated with 6-hydroxy-2,4-alkadienal by LC-MS. Mass spectrum of εGua (**a**) and LC-MS profiles (SIM by molecular ion 176 and fragment ion 148, **b**-**g**). **b**, **c** εGua standard, **d**, **e** reaction of HDE-2 with double-strand-DNA, **f**, **g** reaction of DDE-2 with double-strand-DNA

All four compounds (HDE-1, HDE-2, DDE-1, and DDE-2) were tested for the formation of ε − Gua in ss- and double-stranded (ds-) DNA. Its formation in DNA was confirmed by LC-MS in all samples. Figure 18 shows typical examples of LC-MS chromatogram (SIM). The calibration curve for the peak area and concentration of εGua is shown in S2. The concentration of *εGua* (ng/mL) in DNA (475 µg/mL) is shown in the Table 1. Although all data cannot be compared directly because concentrations between 6-oxo- and 6-OH-compounds differ, when comparing the ratio of ε − Gua yield between ss- and ds-DNA (in terms of ss-DNA/yield in ds-DNA, s/d), the formation ratio was in the order, HDE-1 (s/d = 13) > DDE-1 (s/d = 6) > HDE-2 (s/d = 3) > DDE-2 (s/d = 0.5). 6-oxo-compounds showed higher reactivity with ss-DNA than ds-DNA, while in 6-OH-compounds, the difference in reactivity with ss- and ds-DNA was ambiguous.

**Table 1:** Formation of εGua in ss- and ds-DNA [Mean ± SD (ng/mL)]

	ds-DNA	ss-DNA	ssDNA/dsDNA
Formation of ethenoGua in DNA by 6-oxo-compounds			
6-oxo-2,4-hepadienal	0.65 ± 0.02	^a^8.40 ± 0.04	12.9
6-oxo-2,4-decadienal	0.44 ± 0.01	2.64 ± 0.03	6.0
Formation of ethenoGua in DNA by 6-hydroxy-compounds			
6-OH-2,4-hepadienal	1.39 ± 0.05	^b^4.12 ± 0.09	3.0
6-OH-2,4-decadienal	2.17 ± 0.05	0.97 ± 0.04	0.45

^a^8.90 εGua/10^5^Gua

^b^4.35 εGua/10^5^Gua

## Discussion

Several studies have reported the formation mechanisms of εGua in nucleosides and DNA by lipid peroxidation products, such as 4-OH-2,3-epoxy-nonanal, 4-OOH-compound, 4,5-epoxy-2-decenal, etc. [20, 24–26]. However, in those studies, conditions that differ from in vivo situations, such as pH 9.4, 50 °C, were often used, and the yields were relatively low [24]. In the present study, whole heated oil was directly reacted with the guanine derivative ipG as a new approach. The minor adduct 8-OH-ipG seems to be an ubiquitous adduct in heat-processed foods because, in previous experiments, heated glucose or starch efficiently generated 8-OH-ipG from ipG [12, 13]. Detection of εGua without a side chain as the major adduct by heated cooking oil was unexpected and surprising because many lipid peroxidation products are known to interact with Gua, forming various Gua-adducts [27]. In whole heated oil, εGua may be formed by the precursors 6-oxo- and 6-hydroxy-2,4-alkadienal and 4,5-epoxy-2-alkenal, in the presence of organic hydroperoxyl compounds in heated oil, with a concerted action (mechanism). It is worth mentioning that the reported concentration of 4,5-epoxy-2-decenal (0.86–8.02 μg/g, by heating 3 min to 6 h at 170 °C) in heated oil [28] and amount of 6-hydroxy-2,4-alkadienal (3.8–4.6 μg/g, by heating 45 min at 170 °C) detected in the present study are comparable (within the same range, same order). In addition, 4,5-epoxy-2-heptenal was decomposed in acidic gastric conditions, while 6-oxo- and 6-hydroxy-2,4-heptadienal were not, suggesting that the latter is more important for the induction of human cancer. 6-oxo- and 6-hydroxy-2,4-alkadienal may be produced either by oxidation of 2,4-alkadienal (Fig. 10, right pathway) or via bishydroperoxide formed from linoleate and linolenate (Fig. 10, left pathway) [29] by heating of cooking oil at 170 °C. 6-Oxo- and 6-hydroxy-2,4-heptadienal were also efficiently produced by 2,4-heptadienal auto-oxidation under physiological conditions (pH 7.4, 37 °C), suggesting they could be formed within cells. By combining hemin and tBuOOH, their formation from HDE was accelerated. It can be suspected that they are formed during food digestion in the stomach (acidic pH) and intestine (neutral pH) after meals of meat and fried foods because heme is partially dissociated from myoglobin [30, 31], and high concentrations of hydroperoxide compounds are generated in the stomach and intestine [32, 33]. It is worth mentioning that sequential treatment of cultured cells with hemoglobin and linoleic acid hydroperoxide resulted in an increased εGua level in the DNA [33].

**Fig. 19:**
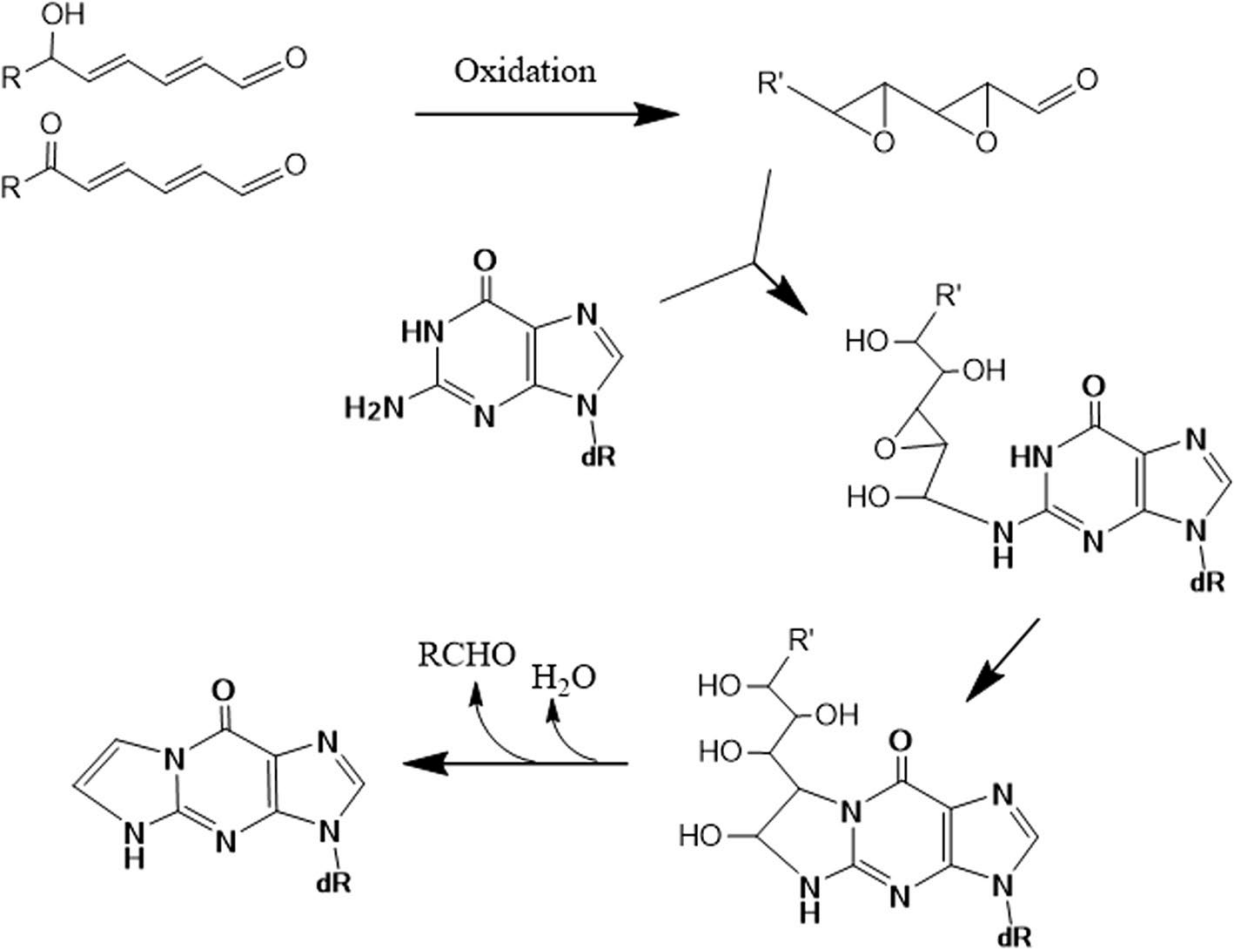
Scheme of mechanism for εGua formation (1), based on the proposal by Loureiro et al. [24]

**Fig. 20:**
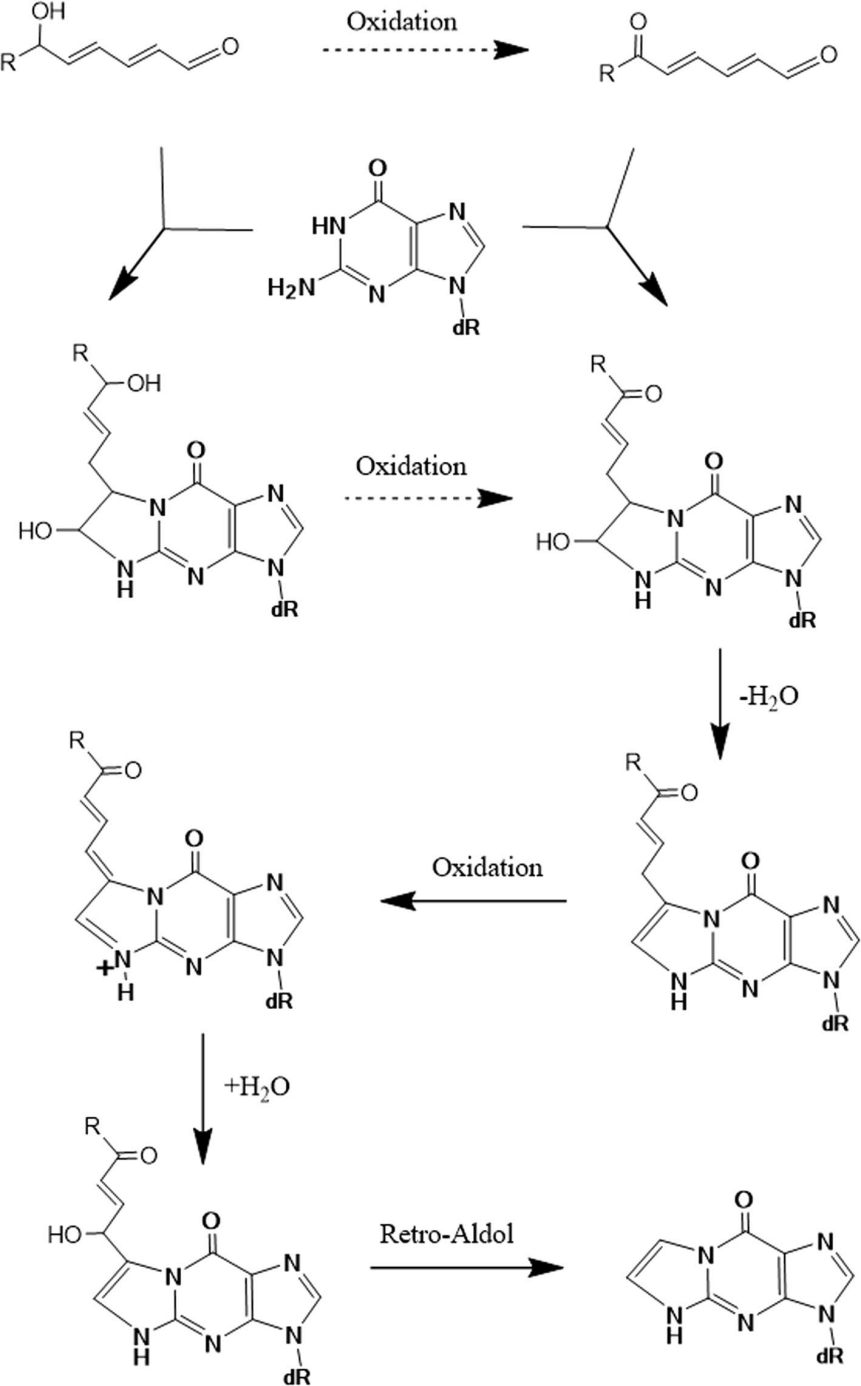
Scheme of mechanism for εGua formation (2), based on the proposal by Petrova et al. [25]

Various pathways are possible for forming εGua from Gua using two precursors. In Figs. 19 and 20, two mechanisms based on those proposed by Loureiro et al. [24] and Petrova et al. [25], for εGua formation by DDE and 4,5-epoxy-2-decenal, respectively, are shown. In both pathways, oxidation steps are required. 6-Oxo- or 6-hydroxy-group may facilitate these oxidation steps. In Fig. 19, after the initial epoxidations at 2,4-dienal, 1,N^2^-cyclization, the elimination of water, and loss of side chains may occur. In Fig. 20, after the initial 1,N^2^-cyclization, the latter oxidation steps may facilitate the final loss of the side chain by retro-aldol reaction.

Small etheno-type adducts, such as εGua has strong mutagenic potential, particularly in mammalian cells, while bulky substituted etheno-type and propano-type cyclic adducts block replication and transcription and are lethal [34]. εGua is a major product formed in DNA exposed to ethyl linoleate or 4-hydroxynonenal under peroxidizing conditions in vitro [35]. A considerable increase in εGua was also observed in response to chronic inflammation in a mouse model [36]. Reportedly, εGua in DNA is mutagenic in *E. coli* and mammalian cells [26, 37], which induces miscoding by human DNA translesion polymerase η in an error-prone manner [38].

Grootvelt et al. pointed out that adverse health effects associated with the intake of toxic lipid oxidation products, such as malonaldehyde, 4-hydroxynonenal, acrolein, and 2,4-alkadienal, which are present in fried food at high concentrations, are not receiving significant attention compared to acrylamide [39]. Higher levels of 2,4-alkadienal have been detected in human plasma from congestive heart failure patients [39] and in relation to cigarette smoking and alcohol consumption [40].

The results of our study may partly explain how fried foods induce human cancer, especially gastrointestinal cancer. Further studies, such as analysis of εGua in gastrointestinal tissue DNA after ingestion of heated cooking oil in animals, or epidemiological studies on the relationship of εGua levels in human tissue DNA or urine with dietary habits of fried foods, are required to confirm this carcinogenesis mechanism. By identifying the causative agents in heated cooking oil, it is also possible to provide strategies for cancer prevention by trapping or decomposing these precursors.

### Supplementary Information

**Additional file 1: S1.** High-resolution mass spectra of HDE-1, HDE-2, DDE-1, and DDE-2. **S2.** Calibration curb for peak area and concentration of εGua.

## Data Availability

Not applicable.
