# Supplementary figures and images for: Formation of the mutagenic DNA lesion 1,N2-ethenoguanine induced by heated cooking oil and identification of causative agents
Source: Genes Environ. 2023 Oct 25;45:27. doi: 10.1186/s41021-023-00284-3 (PMC10599067; doi:10.1186/s41021-023-00284-3)

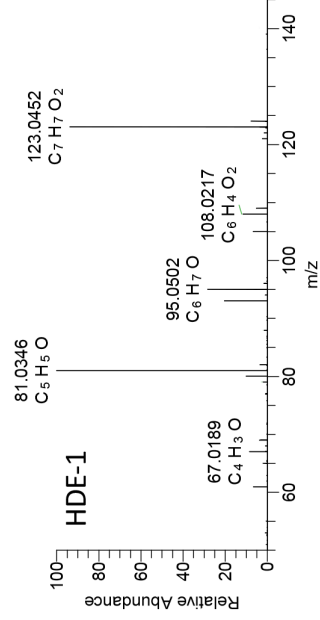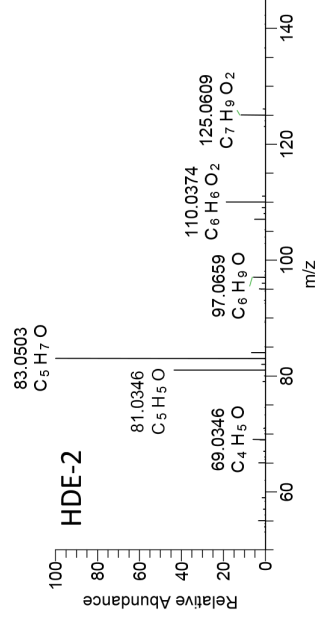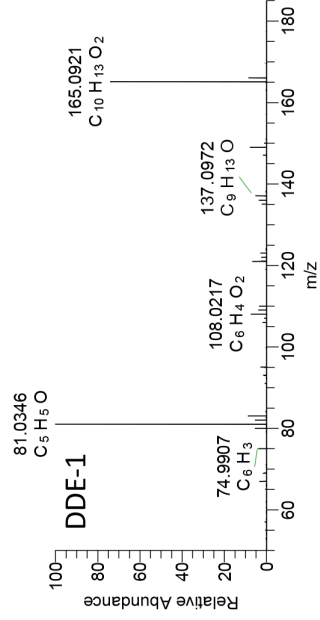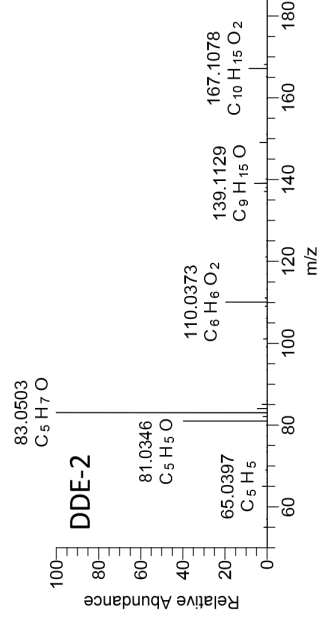

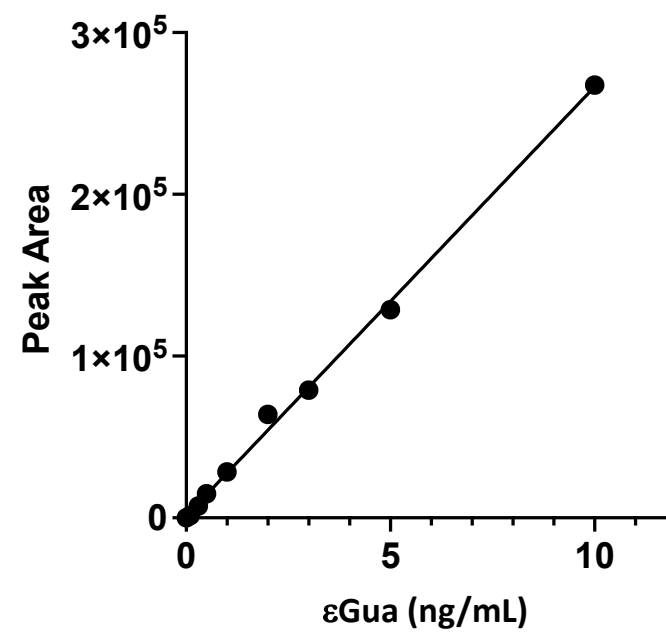

Supplement: Supplementary file 1 — Additional file 1: S1. High-resolution mass spectra of HDE-1, HDE-2, DDE-1, and DDE-2. S2. Calibration curb for peak area and concentration of εGua. [file 41021_2023_284_MOESM1_ESM.pdf]
